# Physicochemical Fingerprint of “Pera Rocha do Oeste”. A PDO Pear Native from Portugal

**DOI:** 10.3390/foods9091209

**Published:** 2020-09-01

**Authors:** Soraia I. Pedro, Elisabete Coelho, Fátima Peres, Ana Machado, António M. Rodrigues, Dulcineia F. Wessel, Manuel A. Coimbra, Ofélia Anjos

**Affiliations:** 1 IPCB, Instituto Politécnico de Castelo Branco, 6001-909 Castelo Branco, Portugal; soraia_p1@hotmail.com (S.I.P.); fperes@ipcb.pt (F.P.); amrodrig@ipcb.pt (A.M.R.); 2 CEF, Centro de Estudos Florestais, Instituto Superior de Agronomia, Universidade de Lisboa, 1349-017 Lisbon, Portugal; 3 LAQV/REQUIMTE, Departamento de Química, Universidade de Aveiro, 3810-193 Aveiro, Portugal; ecoelho@ua.pt (E.C.); ferdulcineia@esav.ipv.pt (D.F.W.); mac@ua.pt (M.A.C.); 4 LEAF, Instituto Superior de Agronomia, Universidade de Lisbon, 1349-017 Lisbon, Portugal; 5 SONAE MC, Sonae MC Serviços Partilhados S.A., 4470-177 Maia, Portugal; AMSILVA@sonaemc.com; 6 CERNAS Research Centre, Polytechnic Institute of Castelo Branco, 6001-909 Castelo Branco, Portugal; 7 School of Agriculture, Polytechnic Institute of Viseu, Quinta da Alagoa—Estrada de Nelas, Ranhados, 3500-606 Viseu, Portugal; 8 CITAB, Centre for the Research and Technology of Agro-Environmental and Biological Sciences, University of Trás-os-Montes and Alto Douro, 5001-801 Vila Real, Portugal; 9 Centro de Biotecnologia de Plantas da Beira Interior, 6001-909 Castelo Branco, Portugal

**Keywords:** “Rocha” pear, Protected Designation of Origin, chemical composition, quality, near infrared (NIR)

## Abstract

“Pera Rocha do Oeste” is a pear (*Pyrus communis* L.) variety native from Portugal with a Protected Designation of Origin (PDO). To supply the world market for almost all the year, the fruits are kept under controlled storage. This study aims to identify which classical physicochemical parameters (colour, total soluble solids (TSS), pH, acidity, ripening index, firmness, vitamin C, total phenols, protein, lipids, fibre, ash, other compounds including carbohydrates, and energy) could be fingerprint markers of PDO “Pera Rocha do Oeste”. For this purpose, a data set constituting fruits from the same size, harvested from three orchards of the most representative PDO locations and stored in refrigerated conditions for 2 or 5 months at atmospheric conditions or for 5 months under a modified atmosphere, were selected. To validate the fingerprint parameters selected with the first set, an external data set was used with pears from five PDO orchards stored under different refrigerated conditions. Near infrared (NIR) spectroscopy was used as a complementary tool to assess the global variability of the samples. The lightness of the pulp; the b* CIELab coordinate of the pulp and peel; and the pulp TSS, pH, firmness, and total phenols, due to their lower variability, are proposed as fingerprint markers of this pear.

## 1. Introduction

**Figure 1:**
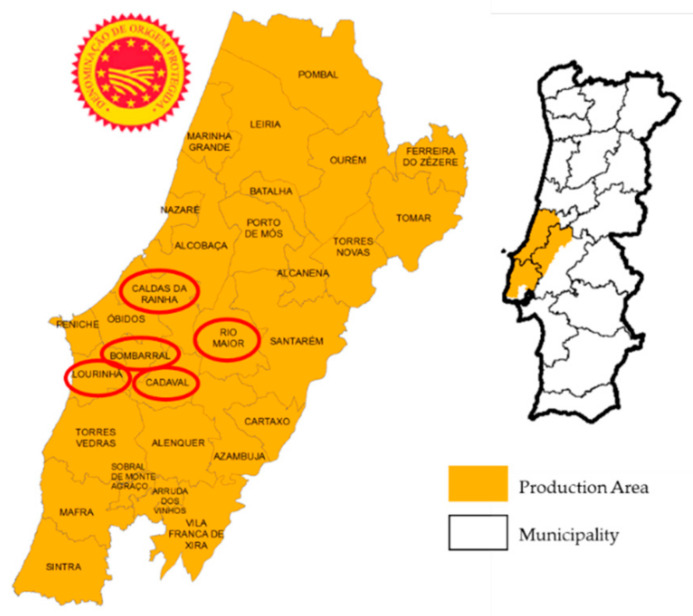
The “Pera Rocha do Oeste” Protected Designation of Origin (PDO) pear region in Portugal. The orchards sampled have the location surrounded with a red circle [4].

“Pera Rocha do Oeste” is a pear (*Pyrus communis* L.) variety with a PDO (Protected Designation of Origin) and one of the most relevant Portuguese fruit products, both in terms of area (12,000 ha) and of economic and export importance (141,186 ton) [1]. The quality attributes of “Pera Rocha do Oeste” include its firmness, acidity, soluble solids content, colour, volatile compounds, absence of defects or imperfections in the epidermis, and the existence of a stem [2]. This fruit presents a crisp texture, high digestibility, as well as a high content of phenolic compounds with antioxidant properties [2]. “Pera Rocha do Oeste” consumers’ acceptance is due to its organoleptic properties, mainly related to taste and firmness [3]. The Ribatejo and Oeste regions of Portugal, in the western region of Portugal, stand out as the most important “Pera Rocha do Oeste” pear-producing areas, both in terms of the number of farms and the cultivated area. The municipalities of Bombarral, Cadaval, Caldas da Rainha, and Lourinhã (Figure 1) are the most representative, with orchards that cover about 70% of the total area and production.

Pears, a climacteric fruit, are harvested mature but unripe and can be ripened off the plant, remaining physiologically active in the post-harvest phase. Along with ripening, the decrease in firmness, acidity, and phenolic compounds and the increase in the content of soluble solids and volatile compounds are changes essentially regulated by the internal production of ethylene, together with an increase in respiratory rate [2,5]. There are several different types of conditions for pear storage. The most common in Portugal are the normal atmosphere (NA) (20–21% O_2_, 0.03% CO_2_, 78–79% N_2_, and a set of other trace gases) [6]; the controlled atmosphere (CA) (using a combination of refrigeration temperature (−0.5 °C), high relative humidity (95%), and control of the partial pressure of atmospheric gases inside the chambers: O_2_ (2–3 kPa) and CO_2_ (0.5–0.7 kPa)) [5,7]; and the dynamic controlled atmosphere (DCA), where the concentration of O_2_ is gradually reduced to the lower limit tolerated by the fruit (less than 1 kPa) [8]. While in a normal atmosphere the fruits can only be kept for a maximum period of 5 months, in a dynamic controlled atmosphere they can be kept for up to 10 months [3,5,6,9]. During storage, the refrigeration temperature must be adequate to reduce the ripening speed, production of ethylene, respiration rate, and consequent changes in the microbial activity. Each product has an optimum storage/preservation temperature [8,9], and pears have a storage temperature that varies between −0.5 and 1 °C [9]. The control of the relative humidity of the air is another decisive factor in long storage, since it reduces the loss of water in the fruit tissues. To preserve pears, a relative humidity between 90% and 95% is used [9]. Storage in controlled or dynamic controlled atmosphere allows maintaining the quality of the fruits for prolonged periods, delaying the ripening and senescence of the fruits [10]. These atmospheres are used, at industrial scale, for the pear fruits that will be consumed after prolonged refrigeration times.

Near infrared (NIR) spectroscopy has been applied for the evaluation of the quality of many foods and beverages given its simplicity, fast analysis, and the fact that it can be non-destructive. The NIR technique has been successfully used in different foods and beverages [11,12,13] to measure different parameters of pear and apple quality attributes and nutraceutical properties [14,15,16]. NIR technology has been mostly used to measure the total soluble solids of a variety of fruits [17,18,19]. Different calibration models were established to evaluate the predictive effect on the quality of different fruits [19,20,21]. To date, there are few reports concerning the applicability of NIR in pear studies. However, concerning the nutritional characterization of the Rocha pear and the limits of the physicochemical parameters that characterize the fruits belonging to the PDO region, the studies are scarce.

In the present study, it is hypothesized that the simple and easy-to-obtain physicochemical parameters, usually used to measure the quality of fruits, can be used as fingerprint markers of “Pera Rocha do Oeste” independently of the storage conditions and orchard of production within the PDO region. To fulfil this novel approach, a data set of sized fruits from three orchards stored in refrigerated conditions for 2 or 5 months at atmospheric conditions or for 5 months under a modified atmosphere was used to identify the parameters with a lower variability. To validate the selected fingerprint parameters, a second data set was used with pears from five PDO orchards stored under different refrigerated conditions. As a complementary tool to assess the global variability of the samples, NIR spectroscopy was used.

## 2. Materials and Methods

### 2.1. Samples

After being received in the laboratory, the fruits were stored at 4 °C, no more than 4 days, until further analysis. Two different data sets were used.

In the first one (set1), 36 “Pera Rocha do Oeste” pear samples (*Pyrus communis* L.), 2018 harvest, were used to identify the characteristic physicochemical parameters independently of the natural variability given by the orchard or the storage method applied to the fruit. Fruits were selected from three different orchards: Caldas da Rainha—Orchard 1 (O1); Lourinhã—Orchard 2 (O2); and Rio Maior—Orchard 3 (O3) (Figure 1). At harvest, the fruits had the following characteristics: size of 50–65 mm, firmness of 5.7–6.9 kg/cm^2^, and total soluble solids (TSS) of 10.7–13.6%, values that were in accordance with European regulations (Reg. (EEC) No. 920/89, of 10 April for PDO “Pera Rocha do Oeste”) [22]. The fruits were stored in two different regimes: refrigerated atmosphere (RA) with 21% O_2_, 0.5% CO_2_, temperature of −0.5 °C, and relative humidity of 95% for two months (RA2) and 5 months (RA5); and dynamic controlled atmosphere (DCA) with 0.7% O_2_, 0.5% CO_2_, temperature of −0.5 °C, and 95% relative humidity for 5 months. To mimic market conditions, DCA was not applied for a 2 months storage time, as this condition is only used for fruits commercialized with a higher storage time.

In the second step (set2), 36 “Pera Rocha do Oeste” pear samples, from five orchards, were used to validate that the physicochemical parameters could be identified as a fingerprint by the first dataset. Pear samples were obtained from the 2018 and 2019 harvests from orchards from Caldas da Rainha, Lourinhã, Rio Maior, Cadaval, and Bombarral. Regarding the storage methods, the aforementioned pears came from the RA2 and RA5 storage methods.

### 2.2. Nutritional Parameters

A “Pera Rocha do Oeste” pear sample nutritional analysis was carried out. Ash, crude fat, and crude protein (Kjeldahl N X 6.25) were determined following the standard method [23], while the carbohydrate contents were calculated by difference (100-crude protein-crude fat-ash-crude fibre). Before the evaluation of nutritional parameters, the pear samples were dehydrated for 24 h at 65 °C.

Crude fat was determined by extraction from the dried sample using petroleum ether b.p. 40–60 °C (CAS number: 8032-32-4; Sigma-Aldrich) as an organic solvent in a Soxtec System HT1043 Extraction unit apparatus (Tecator, Hoganas, Sweden). In the Kjeldahl method for crude protein content evaluation, the sample was firstly digested (Digestion System 6 (1007)—Tecator, Hoganas, Sweden) with concentrated sulfuric acid. The catalysts used were copper sulphate (CAS number: 7758-98-7) + potassium sulphate (CAS number: 7778-80-5); boric acid (CAS number: 10043-35-3) with indicators; 40% sodium hydroxide (CAS number: 1310-73-2) and 0.1 M hydrochloric acid (CAS number: 7647-01-0). All were purchased from Sigma-Aldrich. The mixture was distilled using the Kjeltec System 1026 Distilling Unit—Tecator (Tecator, Hoganas, Sweden).

Crude fibre was determined by the Weende method (Fibertec System1020, Tecator) [23]. This method consists in an oxidative hydrolytic degradation of the cellulose and lignin by sulfuric acid solution (0.13 M sulfuric acid solution; CAS number: 7664-93-9, purchased from Supelco) and subsequent alkali (0.32 M sodium hydroxide; CAS number 1310-73-2 from Supelco) treatment is performed. The residue obtained after filtration and incineration gives the crude fiber content. The energy value was calculated according Regulation (EU) No. 1169/2011 [24].

### 2.3. Quality Parameters

The fruits were characterized by their total soluble solids (TSS), color, ascorbic acid content, pH, weight, titratable acidity, total phenols, and firmness. Fruits from each region were selected and analyzed in triplicate.

Total soluble solids (%) were evaluated by refractometry with a digital refractometer (Hanna Instruments HI 96801, Hanna Instruments Portugal, Póvoa de Varzim, Portugal).

The color of pears was measured by a Minolta Chromameter CR 300 (from Minolta, Tokyo, Japan). The results were obtained in CIELAB (L*, a*, b*) color space. L* defines lightness, while a* and b* define the red-greenness and blue-yellowness, respectively. A white tile (L* = 97.10; a* = 0.08; b* = 1.80) was used as a reference. The samples were analyzed on the epidermis (peel) and the respective mesocarp (pulp) (four locations for each sample).

To perform the evaluation of the ascorbic acid content, the 2,6-dichloroindophenol titrimetric method was used [23,25], which is considered the official method of analysis for vitamin C. In this method, the vitamin C in pear juice was determined by oxidizing it in acid medium with 2,6-dichlorophenol indophenol (CAS number 1266615-56-8) to dehydroascorbic acid. The titer of the dye was determined by using a standard ascorbic acid (CAS number: 113170-55-1) solution (1 mg ascorbic acid/g solution, dissolved in 2% (*w/v*) metaphosphoric acid (CAS number: 37267-86-0) solution). The results were expressed as the mg of ascorbic acid/100 mL of the sample.

The titratable acidity was measured by a potentiometric titrator (Metrohm 702 SM Titrino, Herisau, Switzerland) after the titration of 10 mL of pulp juice against 0.1 M of NaOH until pH 8.15 was reached. The results were expressed as malic acid equivalents (g/100 mL). With the ratio of the TSS and titratable acidity, the ripening index (RI) was calculated for all the samples.

The extraction of total phenols present in “Rocha” pears was performed following the method described by Serra et al. [26] The extraction of polar compounds was performed by the addition of 150 mL of acetone (80% (*v/v*)) to 50 g of pear, followed by centrifugation at 5000 rpm for 10 min. The total phenols were determined according to the modified Folin–Ciocalteau colorimetric method at a 765 nm wavelength (spectrophotometer Jasco UV/VIS 7800; Jasco, Tokyo, Japan), and the results expressed as gallic acid equivalents (mg GAE/100 g). Gallic acid standard (CAS number: 149-91-7) was purchased from Sigma-Aldrich (city, state, country).

Firmness was determined by a penetration test at room temperature (20 ± 2 °C) on a texturometer by a texture analyzer (CT3 Texture Analyzer Brookfield, Germany). The results were expressed as the maximum force in Newtons (N), which was recorded by a 2 mm diameter probe (TA-MTP-3R) penetration into the pear tissue to 10.0 mm (a small section of each fruit was removed to give a stable test surface through which to penetrate). The force–distance curves were recorded and the firmness was taken as the maximum force peak (N) of the curves. The test speed was 0.50 mm/s.

### 2.4. NIR

On each pear (surface of the intact fruit), four reflectance spectra in four different positions were acquired with an NIR spectrometer (MPA Bruker, Germany) using reflectance light. The samples were measured with a spectral resolution of 8 cm^−1^ and 32 scans in the wavenumber from 12,000 to 4000 cm^−1^.

### 2.5. Statistical Analysis

A standardized biplot of principal component analysis (PCA) was carried out, aiming at an overall evaluation of the chemical data and factors effect (storage method and orchard), which displays the standardized component scores and the standardized variable projections. The PCA was performed for data set1 and data set2.

Classification trees were used in this work for set1 to understand which variables are more representative to differentiate—Firstly, by the storage method used and, in a second analysis, by orchards from the same PDO region. At each step, the most informative parameters were selected as the root of the (sub)tree, and the current training set was split into subsets according to the values of the selected attribute. It was considered a good discriminator parameter if the branches separated all the measurements observed for each attribute. Number 4 was selected for nodes. After a variable was selected, the classification tree algorithm was run again without this parameter to understand if there was other parameter that could also be used to distinguish the attributes. The chemical parameters not identified by the classification trees as discriminators for storage or orchard were selected as possible fingerprint markers of the “Pera Rocha do Oeste” pear.

PCA and classification trees analysis were performed using Statistics^®^ version 7.0. To perform PCA with spectral data, different mathematical transformation techniques were used—namely, Savitzky–Golay first and second derivative, standard normal variate (SNV) transformation, multiplicative scatter correction (MSC), and different combinations of these treatments. The Unscrambler^®^ X software, version 10.5.46461.632 (CAMO Software AS, Oslo, Norway), was used for the spectral data analyses.

## 3. Results and Discussion

### 3.1. Influence of Storage Conditions and Orchard Origin of “Pera Rocha do Oeste” on Its Physicochemical Characteristics

To study the influence of the storage conditions and orchard origin of “Pera Rocha do Oeste” on its physicochemical characteristics, a data set of pears from three orchards and stored under three different storage conditions (RA2—refrigerated atmosphere for 2 months; RA5—refrigerated atmosphere for 5 months; DCA—dynamic controlled atmosphere for 5 months) was used (set 1). 

The analysis of the CIElab color data obtained for the pear peel and pulp revealed a natural variability of the fruits, with a higher variation in the a* and b* parameters (Table 1). The observed a* and b* values for the pear peel ranged from −12.87 to 5.64 and 39.17 to 45.52, respectively. For the pulp, the a* and b* values ranged from −11.07 to 2.25 and 8.15 to 17.68, respectively. Similar color values for Rocha pear pulp were reported in other studies [27,28]. The a* and b* values of peel are considered good indicators to measure the influence of storage conditions [27], presenting values able to diagnose the storage of the fruits.

The weight loss of fruit and vegetables during storage occurs due to the loss of water from tissues related to transpiration, as well as respiration, which contributes also with the release of carbon dioxide, resulting in tissue shrinking and textural changes, with the consequent loss of marketability [6]. Pear storage in a chamber with a controlled atmosphere with a higher humidity is intended to prevent the aforementioned effect. The storage conditions were effective in the preservation of fruit weight, where water and weight loss did not occur (data not shown), which were kept in the values of 81.90 ± 6.71%, maintaining the total soluble solids (TSS) and titratable acidity (TA) for as long as possible. Table 2 shows that TSS have an average value of 11.94 ˚Brix, ranging from 13.80 to 10.70 ˚Brix, explained by the different ripening stages of the fruits. Titratable acidity presents average values of 0.20 ± 0.08 g malic acid equivalents/100 mL (Table 2). The sugar and acidity content is important for fruit taste [29], as fruit sweetness is related to sugar content modulated by acidity. The ripening index, given by the ratio between the total soluble solid content and the titratable acidity (TSS/TA), is an expression of taste equilibrium. Fruits with low ratio values are perceived as acidic, while too high values are equally unpleasant. This index ranged from 37.24 to 141.11. The TSS and TA values observed in this work were similar to those reported for the “Pera Rocha do Oeste” pear [8,28], as well as those for Bartlett pear [30] and a *Pyrus communis* L. variety.

Considering the pH values (Table 2), the “Pera Rocha do Oeste” pear can be classified as acidic, with a value of 4.81 ± 0.08, in accordance with the literature [28]. The total phenolic compounds, ranging between 39.26 and 93.10 mg gallic acid equivalents/100 g, are also similar to the literature [21]. The vitamin C content ranged between 2.08 and 10.42 mg ascorbic acid/100 mL. These values are characteristic of this cultivar, taking into account that the pear has a low vitamin C content [31,32]. The firmness of the “Pera Rocha do Oeste” pear under study had average values of 12.21 ± 2.51 N, which are similar to the literature [27,28]. The fruit flesh firmness decreases over time due to metabolic processes by the depolymerization of the cell wall polysaccharides, with a decrease more pronounced for pears undergoing 2 months of storage [33]. However, this trend was not observed in the present work due to the cold storage performed, minimizing the metabolic activity. According to the obtained values for the physiochemical parameters, the “Pera Rocha do Oeste” pear variety presented an excellent storage capacity for up to 5 months under controlled conditions, as previously reported [27].

Table 3 summarizes the results of the nutritional composition of the fruits for the different orchard and for the fruits submitted to the different storage methods. The protein and lipid contents were, on average, 2.07% ± 0.23% and 0.36% ± 0.04%, respectively. The “Pera Rocha do Oeste” pear showed a fibre content that ranged from 8.14% to 13.55%. These results are in accordance with the literature [34]. Concerning the ash content, the observed values ranged between 1.44% and 2.91%. Similar values were found by [34]. The parameter “Other”, which included carbohydrates, referring to sugars and starch, ranged between 7.60% and 29.12%. These values were higher for the pear stored for longer periods. Although carbohydrate content plays an important influence during the storage process, in the case of the “Pera Rocha do Oeste” pear, the fruit firmness was not so affected, as already stated in the literature [35]. In addition, the pears under study are characterized by an energy content of 19.85 ± 2.46 kcal/100 g, with values between 15.77 and 24.83 kcal/100 g.

### 3.2. Identification of Fingerprint Parameters

**Figure 2:**
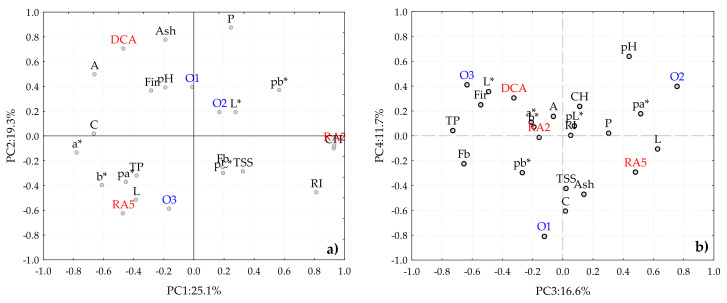
Principal component analysis biplot (scores and loadings) for set1, considering the different orchard and storage method: (**a**) PC1 vs PC2; (**b**) PC3 vs PC4. A—Titratable acidity; Ash—Ash; C—Vitamin C; CH—other including carbohydrates; CIELab coordinates for pear peel L*, a*, b*; CIELab coordinates for pear pulp pL*, pa*, pb*; DCA—dynamic controlled atmosphere for 5 months; Fb—fibre; Fir—Firmness; Li—Lipids; O1, O2, and O3 represent 3 different orchards; P—protein; RA2—refrigerated atmosphere for 2 months; RA5—Refrigerated atmosphere for 5 months; RI—Ripening index; TP—Total phenols; TSS—Total soluble solids.

The PCA performed with the physicochemical characteristics of “Pera Roca do Oeste” pears grouped in data set1 explains at least 72.7% of the total variation with four Principal Components (PC1 = 25.1%; PC2 = 19.3%; PC3 = 16.6%; PC4 = 11.7%). Figure 2 presents the PC1 vs. PC2 biplot (scores and loadings), considering the different orchards and storage methods. A clear differentiation is observed between the studied storage methods, mainly between the pears stored for 2 and 5 months. It is also evident that some variables are less influenced by the storage method and, consequently, are candidates to be used to fingerprint the “Pera Rocha do Oeste” pear. Along with PC1, there were no observed differences between the orchards, allowing us to conclude that the storage conditions provide a much higher variability in physicochemical parameters than the orchard location within the PDO region.

**Figure 3:**
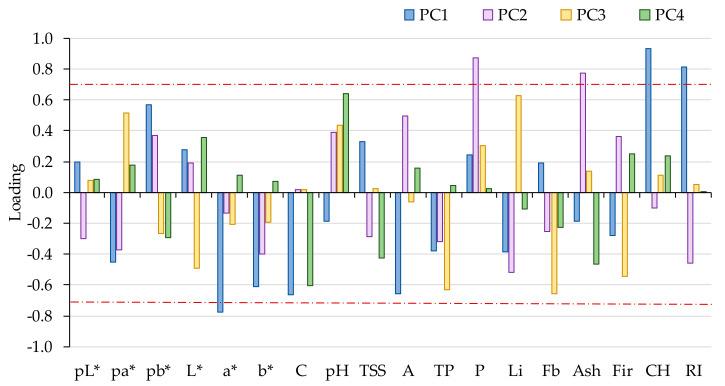
Schematic representation of the loadings of the three first components of the PCA, considering the different orchard and storage methods. A—Titratable acidity; Ash—Ash; C—Vitamin C; CH—other including carbohydrates; CIELab coordinates for pear peel L*, a*, b*; CIELab coordinates for pear pulp pL*, pa*, pb*; P—Protein; RI—Ripening index; TP—Total phenols; TSS—Total soluble solids.

The loading factors that represent the correlation between the physicochemical variables and the principal components were used to classify the dominant variables in each principal component that characterizes the “Pera Rocha do Oeste” PDO pear. The acceptable threshold of 0.7 (absolute value) can be considered as dominant, playing a main role in that principal component [36]. As an analytical parameter that does not vary with the orchard and the storage method can be a good candidate for a fingerprint parameter to characterize the PDO pear, a graphic with the loadings for the four first components for each parameter was performed (Figure 3), and the values of 0.7 and −0.7 were marked as the lower and upper limits.

According to Figure 3, the physicochemical parameters that can be promising to fingerprint the “Pera Rocha do Oeste” pear are the L*, a*, and b* CIELab coordinates for the pear peel color; the L* and b* CIELab coordinates for the pear pulp color; C; pH; TSS; A; TP; Li; Fb; and Fir.

The fruits stored two months (RA2) were placed in PC1 positive, whereas the pears stored for five months (RA5 and DCA), independently of the method, were placed in PC1 negative, distinguished mainly by the concentration of carbohydrates, classified as “other compounds”, and RI (ripening index) (Figure 2 and Figure 3). Concerning the three orchards, they could not be distinguished in PC1, only in PC2, which explained 19.8% of the total variance, showing that the orchard location within the PDO region is not a high factor of variability considering the physicochemical parameters. To confirm if there could be another important factor that discriminates the storage methods and orchards not identified by PCA, we performed a classification tree to decide if these previously analytical parameters could be considered as fingerprints.

**Figure 4:**
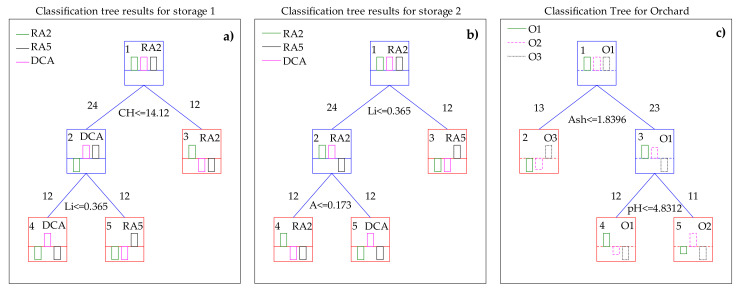
Classification tree results for the storage method and orchard applied to the physicochemical parameters of “Pera Rocha do Oeste” pears using the Gini methodology (**a**) performed with all the analytical parameters for storage; (**b**) performed with all the analytical parameters, excluding CH for storage; (**c**) performed with all the analytical parameters for orchard. A—Titratable acidity; CH—other including carbohydrates; DCA—Dynamic controlled atmosphere for 5 months; Li—Lipids; O1, O2, and O3 represent 3 different orchards; RA2—Refrigerated atmosphere for 2 months; RA5—Refrigerated atmosphere for 5 months.

Figure 4 represents the classification tree of the physicochemical data of the “Pera Rocha do Oeste” pear considering their origin from different storage methods and orchards, using the Gini methodology [37]. The first node separated the pears stored for two months (RA2) based on the values observed for the parameter “CH” (other compounds, including carbohydrates) (Figure 4a). The second node separated the fruits based on the lipid content. PCA analyses showed that lipids could be a factor that varied depending on the storage method, considering only PC3 (16.3% of the total variation) (Figure 4b). Based on the classification trees analysis, it was confirmed that lipids cannot be considered as a parameter to fingerprint the “Pera Rocha do Oeste” pear. With the removal of the parameter “CH”, based on the pear acidity it was still possible to separate RA2 and DCA, the pears stored for 5 months under a controlled atmosphere (Figure 4b), which is explained by the higher acidity of the fruits stored in atmospheres enriched in carbon dioxide [38]. Moreover, no other physicochemical variables allowed a clear node separation, allowing us to conclude that carbohydrates (sugars and starch), lipids, and acidity are markers for the pear storage method, and not suitable to be used as fingerprints for the “Pera Rocha do Oeste” pear.

Regarding the classification tree for orchards (Figure 4c), the most important analytical parameter that discriminated the three different orchards was the ash content, a characteristic that can be related to the soil [39]. The pH values also tend to show a separation in the nodes. However, the minimum of 12 samples required was only observed for orchard 1.

Combining the PCA and classification tree analyses, it was possible to confirm the analytical parameters that could be fingerprint markers for the “Pera Rocha do Oeste” PDO pear, because they were parameters not related to the orchard location nor the storage methods usually used to preserve the fruits: the L*, a*, and b* CIELab coordinates for the pear peel color; the L* and b* CIELab coordinates for the pear pulp color; vitamin C; pH; TSS; total phenols; firmness; and fibre content. The other analytical parameters that vary between specific limits and are also characteristic of the “Pera Rocha do Oeste” pear, presenting a variability associated with the storage method or orchard, are a*; A; P; Li; Ash; CH; Fb; and Ri.

### 3.3. NIR Spectroscopy Analysis of the “Pera Rocha do Oeste” PDO Pear

**Figure 5:**
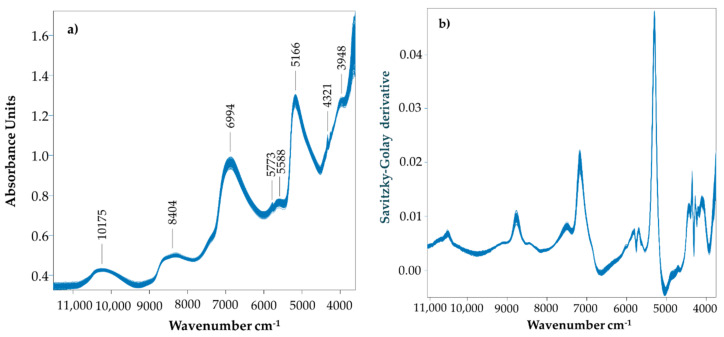
NIR absorption spectra of the “Pera Rocha do Oeste” pear of data set 1 processed with (**a**) multiplicative scatter correction and (**b**) multiplicative scatter correction and the Savitzky–Golay derivative.

The obtained spectra are similar to those reported for pears [40], as well as for different fruits such as acerola [20], apple [41,42], and plum [43]. The NIR spectra of all the pears analyzed had a similar profile, with four main bands around 10175, 8404, 6994, and 5166 cm^−1^ (Figure 5a). The spectral peaks around 5166 cm^−1^ and at 6994 cm^−1^. corresponding to the water absorption bands, can be assigned to the first and second vibrational overtone of -OH stretching [44]. However, these bands can be also associated with carbohydrates, starch, and proteins [45,46]. The bands around 8404 and 5166 cm^−1^ correspond to the 2nd and 1st overtones of -CH stretching, as well as the 3rd overtone of -OH, -CH, and -CH2 deformation associated with sugars [41]. Sugars and organic acids have been reported to display bands in the wavelength regions of 9090–6250 and 5882–4350 cm^−1^ [43,47]. The small peaks at the 5773 and 5588 cm^−1^ regions are also sugar-related and could be assigned by the 1st overtone of the -CH stretch associated also with the presence of sugars [43]. Another small band that appears at 4321 cm^−1^ could also be associated with the C-H stretch and C-H deformation of polysaccharides [48].

**Figure 6:**
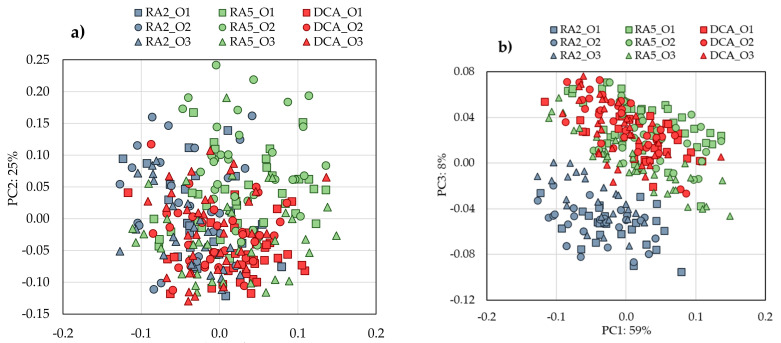
Principal component analysis performed with data set 1 of NIR spectra applying MSC + Savitzky–Golay 1st derivative as a spectral pre-process. (**a**) PC1 vs. PC2 and (**b**) PC1 vs. PC3.

**Figure 7:**
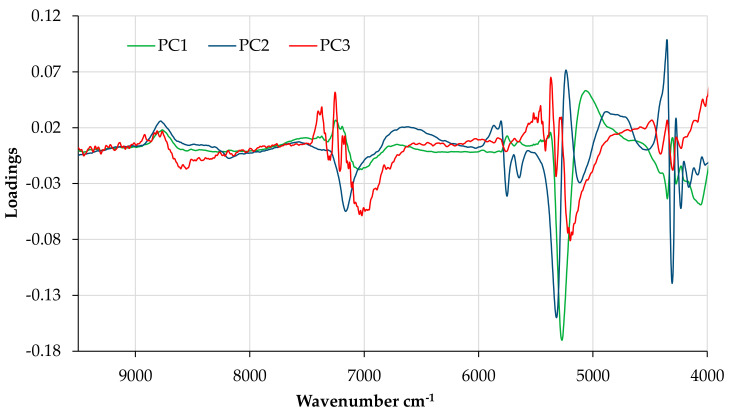
Loading (for the three first components) influence of the principal component analysis performed with data set 1 of the NIR spectra applying MSC + Savitzky–Golay 1st derivative as a spectral pre-process.

Dimensionality reduction was performed by transforming the wavelength variables (Figure 5b) into principal components (Figure 6). Regarding the PC1 vs. PC2 scores plot, the NIR spectra of pears stored in different conditions show a very similar distribution; it is only possible to distinguish in PC3 (8% of the total variation) the pears stored for two months from the ones stored for 5 months in a refrigerated atmosphere and a dynamic controlled atmosphere. These results confirm the consistent physicochemical characteristics of the “Pera Rocha do Oeste” pear. It seems that the classification tree analysis, where it was observed that carbohydrate variation was the most relevant parameter to distinguish the storage of the “Pera Rocha do Oeste” pear, was corroborated by NIR spectroscopy. The more representative NIR region for the differences observed are the peaks at 5200 cm^−1^ for the RA2 samples and the peaks at 4459 and 5300 cm^−1^ for the RA5 and DCA samples (Figure 7). The other spectral region was quite similar for all the samples.

### 3.4. Validation of the Fingerprint Physicochemical Parameters of the “Pera Rocha do Oeste” PDO Pear

**Figure 8:**
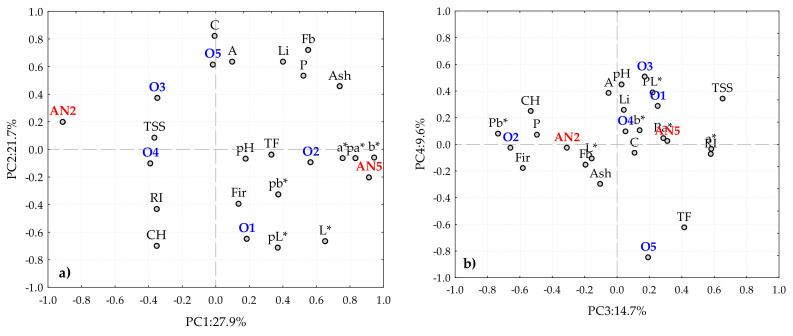
Principal component analysis biplot (scores and loadings), for set2, considering the different orchard and storage methods: (**a**) PC1 vs. PC2; (**b**) PC3 vs. PC4. A—Titratable acidity; Ash—Ash; C—Vitamin C; CH—other including carbohydrates; CIELab coordinates for pear peel L*, a*, b*; CIELab coordinates for pear pulp pL*, pa*, pb*; Fb—Fibre; Fir—Firmness; Li—Lipids; O1, O2, O3, O4 and O5 represent 6 different orchards; P—Protein; RA2—Refrigerated atmosphere for 2 months; RA5—Refrigerated atmosphere for 5 months; RI—Ripening index; TP—Total phenols; TSS—Total soluble solids.

To confirm the aforementioned fingerprint physicochemical parameters of the “Pera Rocha do Oeste” pear, a validation data set was used with fruits from five different orchards and periods of storage. In this data set, fruits from two different years were used and, on average, the measured physicochemical parameters were similar. The PCA performed with the physicochemical parameters of this data set allowed us to obtain four components that accounted for 73.9% of the total variability (PC1 = 27.9%; PC2 = 21.7%; PC3 = 14.7%; PC4 = 9.6%) (Figure 8a,b). The loadings plot (Figure 8) shows a clear differentiation in PC1 between the pears stored for 2 and 5 months and, with a lower variability, differences due to the orchard location, as previously observed in Figure 2 for data set 1.

**Figure 9:**
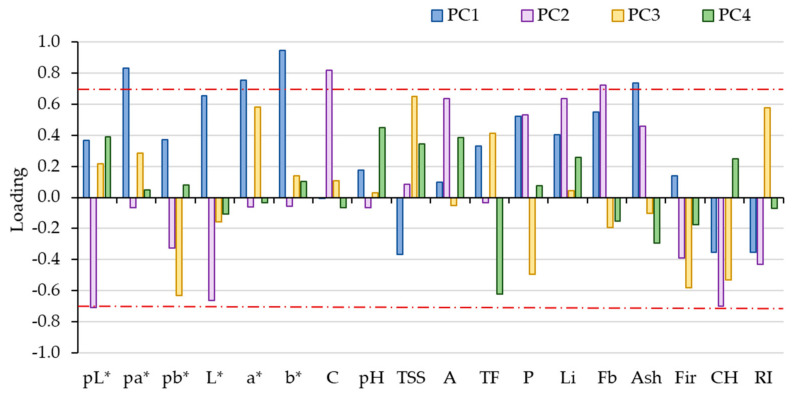
Schematic representation of the loadings of the three first components of the PCA, considering the different orchard and storage methods. A—Titratable acidity; Ash—Ash; C—Vitamin C; CH—other including carbohydrates; CIELab coordinates for pear peel L*, a*, b*; CIELab coordinates for pear pulp pL*, pa*, pb*; Fb—Fibre; Fir—Firmness; Li—Lipids; P—Protein; RI—Ripening index; TP—Total phenols; TSS—Total soluble solids.

Figure 9 represents the loadings for the first four components for each physicochemical parameter obtained for the pears included in the validation data set, allowing us to identify that the pL* and pa* CIELab coordinates for pulp, the b* CIELab coordinate for peel, and vitamin C were dominant factors for data set 2. As a consequence, these parameters could not be included as fingerprint parameters of the “Pera Rocha do Oeste” PDO pear.

The combination of the data obtained with data set 1 and their validation with data set 2 allowed us to conclude that there are physicochemical parameters that do not show relevant variation among the storage of the pears or variation due to their origin within the PDO region—namely, the lightness of the pulp, the b* CIELab coordinate of the peel and pulp (yellow tonality), pH, TSS, total phenols, and firmness.

Table 4 summarizes the intervals of variability that characterize the “Pera Rocha do Oeste” PDO pear physicochemical parameters, obtained with pears with the size of 50–65 mm from seven orchards, harvested in 2018 and 2019, and stored for 2 or 5 months under refrigerated conditions or for 5 months under a controlled atmosphere.

## 4. Conclusions

In the present study, an innovative and practical approach to identify simple and easy to obtain physicochemical markers of the “Pera Rocha do Oeste” PDO pear was proposed based on their stability among the storage of the fruits and the orchard origin. This approach allowed us to propose intervals for the variability of the different physicochemical parameters used to characterize the “Pera Rocha do Oeste” PDO pear. The lightness of the pulp (70.36–87.19), b* CIELab coordinate of the pulp (37.91–47.09), peel (37.91–47.09), pH (4.36–4.92), total soluble solids (10.00–13.80 ˚Brix), total phenols (39.26–93.10 mg GAE/100 g), and firmness (9.10–17.96 N) were the physicochemical parameters that, together, are proposed to be used as fingerprints of the “Pera Rocha do Oeste” PDO pear.

This practical methodology, using physicochemical parameters that are usually measured to characterize the Rocha pear, could be easily implemented by the industry. Additionally, with this research it was concluded that only some analytical parameters must be measured to identify the “Pera Rocha do Oeste” PDO pear, which have the advantage of being less expensive and time consuming.

NIR spectroscopy was used as a complementary tool to recognize the similarities of the “Pera Rocha do Oeste” PDO pear and eliminate those that changed with the storage of the fruits or the origin of the orchards. This study paves the way for future works with more data, allowing calibration models to be performed for an easier identification of the “Pera Rocha do Oeste” PDO pear.

## Figures and Tables

**Table 1:** Color parameters of the “Pera Rocha do Oeste” pear by the orchard, storage method, and global values.

SM	O	Peel			Pulp		
		L*	a*	b*	L*	a*	b*
RA2	1	72.07 ± 2.62	−8.99 ± 2.03	44.07 ± 0.41	80.66 ± 2.64	−9.54 ± 1.63	9.31 ± 1.39
	2	73.49 ± 1.89	−5.72 ± 3.01	43.35 ± 0.48	79.88 ± 1.42	−8.28 ± 1.44	9.08 ± 0.96
	3	73.96 ± 3.17	−10.19 ± 1.90	43.97 ± 1.15	81.25 ± 1.94	−0.77 ± 0.74	13.73 ± 0.82
RA5	1	69.88 ± 1.39	−1.52 ± 2.03	42.60 ± 0.64	77.18 ± 1.57	−0.17 ± 1.61	12.54 ± 0.72
	2	72.37 ± 2.51	3.39 ± 3.01	42.88 ± 0.92	79.07 ± 0.56	−0.33 ± 1.49	14.75 ± 1.97
	3	76.04 ± 0.90	−2.92 ± 1.90	40.88 ± 1.93	79.05 ± 1.25	0.74 ± 1.27	13.63 ± 2.44
DCA	1	72.69 ± 5.84	−7.31 ± 2.94	42.60 ± 1.57	79.33 ± 1.92	−1.87 ± 0.57	11.81 ± 1.72
	2	72.12 ± 1.41	−10.22 ± 2.07	42.88 ± 0.80	78.42 ± 0.88	−1.71 ± 0.14	11.51 ± 1.67
	3	68.49 ± 2.78	−2.92 ± 2.24	40.88 ± 1.58	82.40 ± 3.50	−1.42 ± 0.55	13.03 ± 2.88
μ ± σ		72.34 ± 3.33 66.69; 79.55	−4.79 ± 5.16 −12.87; 5.64	42.40 ± 1.63 39.17; 45.52	79.69 ± 2.60 75.40; 87.19	−2.59 ± 3.60 −11.07; 2.25	12.15 ± 2.46 8.15; 17.68
Min; Max							

SM—Storage method; O—Orchard; RA2—Refrigerated atmosphere for 2 months; RA5—Refrigerated atmosphere for 5 months; DCA—Dynamic controlled atmosphere for 5 months; μ—Average; σ—Standard deviation; Min—Minimum; Max—Maximum.

**Table 2:** Physicochemical parameters of the “Pera Rocha do Oeste” pear by orchard and storage method.

SM	O	TSS (°Brix)	pH	TA (g malic acid/100 mL)	RI	Firmness (N)	Vitamin C (mg/100 g)	Total Phenols (mg GAE/100 g)
RA2	1	12.10 ± 0.12	4.69 ± 0.01	0.13 ± 0.01	94.77 ± 1.53	12.51 ± 1.92	5.42 ± 1.02	65.84 ± 5.49
	2	12.57 ± 0.19	4.91 ± 0.01	0.09 ± 0.01	139.63 ± 2.10	10.15 ± 0.88	2.08 ± 0.59	44.67 ± 6.17
	3	12.57 ± 0.05	4.76 ± 0.00	0.14 ± 0.01	90.19 ± 0.64	11.47 ± 1.61	4.17 ± 0.59	72.33 ± 4.76
RA5	1	13.37 ± 0.31	4.76 ± 0.00	0.22 ± 0.03	61.54 ± 8.18	9.41 ± 0.28	10.42 ± 0.59	66.37 ± 4.74
	2	11.40 ± 0.07	4.91 ± 0.00	0.28 ± 0.01	40.33 ± 0.20	10.59 ± 1.46	5.83 ± 0.51	47.99 ± 3.28
	3	11.23 ± 0.10	4.71 ± 0.0	0.11 ± 0.01	106.57 ± 5.51	13.37 ± 1.36	5.42 ± 0.59	83.95 ± 6.03
DCA	1	11.07 ± 0.45	4.84 ± 0.02	0.29 ± 0.01	38.07 ± 0.62	16.72 ± 0.28	5.83 ± 1.18	58.77 ± 1.21
	2	11.07 ± 0.05	4.84 ± 0.01	0.23 ± 0.02	48.61 ± 3.80	11.11 ± 0.20	7.08 ± 1.56	69.50 ± 7.67
	3	12.10 ± 0.01	4.92 ± 0.00	0.28 ± 0.01	42.62 ± 0.74	14.58 ± 2.12	6.67 ± 0.59	86.48 ± 7.51
μ ± σ		11.94 ± 0.79	4.81 ± 0.08	0.20 ± 0.08	73.59 ± 34.41	12.21 ± 2.51	5.87 ± 2.29	66.21 ± 14.51
Min; Max		13.80; 10.70	4.68; 4.92	0.09; 0.30	37.24; 141.11	9.12; 17.39	1.25; 11.25	39.26; 93.10

SM—Storage method; O—Orchard; RA2—Refrigerated atmosphere for 2 months; TA—Titratable acidity; RI—Ripening index RA5—Refrigerated atmosphere for 5 months; DCA—Dynamic controlled atmosphere for 5 months; μ—Average; σ—Standard deviation; Min—Minimum; Max—Maximum.

**Table 3:** Nutritional parameters of the “Pera Rocha do Oeste” pear by orchard and storage method.

SM	O	Protein (%)	Lipids (%)	Fibre (%)	Ash (%)	Other including Carbohydrates (%)	Energy (kcal/100 g)
RA2	1	2.13 ± 0.05	0.30 ± 0.02	10.03 ± 0.06	2.25 ± 0.04	17.03 ± 0.10	20.21 ± 0.44
	2	2.37 ± 0.0	0.36 ± 0.01	10.43 ± 0.18	1.84 ± 0.02	29.11 ± 0.02	20.04 ± 0.07
	3	1.81 ± 0.01	0.35 ± 0.01	13.01 ± 0.37	1.65 ± 0.03	18.12 ± 0.03	17.00 ± 0.11
RA5	1	2.00 ± 0.01	0.42 ± 0.01	11.31 ± 0.42	2.41 ± 0.08	9.26 ± 0.07	21.42 ± 0.21
	2	2.03 ± 0.01	0.42 ± 0.01	8.40 ± 0.21	1.96 ± 0.02	10.55 ± 0.03	19.73 ± 0.14
	3	1.67 ± 0.03	0.38 ± 0.01	11.33 ± 0.03	1.45 ± 0.01	10.63 ± 0.02	15.84 ± 0.06
DCA	1	2.41 ± 0.08	0.31 ± 0.03	10.68 ± 0.81	2.68 ± 0.21	7.98 ± 0.32	23.11 ± 1.42
	2	2.23 ± 0.02	0.36 ± 0.01	11.09 ± 0.07	2.74 ± 0.04	8.92 ± 0.06	23.11 ± 0.24
	3	2.01 ± 0.04	0.34 ± 0.01	10.10 ± 0.06	1.78 ± 0.04	10.69 ± 0.07	18.17 ± 0.32
μ ± σ		2.07 ± 0.23	0.36 ± 0.04	10.71 ± 1.23	2.08 ± 0.44	13.59 ± 6.52	19.85 ± 2.46
Min; Max		1.64; 2.51	0.27; 0.43	8.14; 13.55	1.44; 2.91	7.60; 29.12	15.77; 24.83

SM—Storage method; O–Orchard; RA2—Refrigerated atmosphere for 2 months; RA5—Refrigerated atmosphere for 5 months; DCA—Dynamic controlled atmosphere for 5 months; μ—Average; σ—Standard deviation; Min—Minimum; Max—Maximum.

**Table 4:** Physicochemical parameter intervals that characterize the “Pera Rocha do Oeste” PDO pear. Mean values, standard deviation (sd), absolute maximum and minimum values. Parameters in bold are those that can be used as fingerprint markers.

Parameter	Mean ± sd	Minimum	Maximum
Peel colour L	71.93 ± 3.82	64.38	80.74
Peel colour a*	−6.75 ± 5.51	−13.57	7.33
Peel colour b*	**41.70 ± 2.29**	**37.91**	**47.09**
**Pulp colour L**	**77.70 ± 3.79**	**70.36**	**87.19**
Pulp colour a*	−3.45 ± 3.58	−11.07	5.11
Pulp colour b*	**11.19 ± 2.94**	**6.81**	**17.68**
Vitamin C (mg/100 mL)	6.37 ± 2.21	1.25	11.25
**pH**	**4.71 ± 0.14**	**4.36**	**4.92**
**TSS (˚Brix)**	**11.69 ± 0.89**	**10.00**	**13.80**
Titratable Acidity (g malic acid/100 mL)	0.16 ± 0.07	0.09	0.30
**Firmness (N)**	**12.33 ± 2.33**	**9.10**	**17.96**
**Total phenols (mg GAE/100 g)**	**64.41 ± 13.35**	**39.26**	**93.10**
Protein (%)	2.21 ± 0.52	1.54	3.51
Lipids (%)	0.38 ± 0.07	0.21	0.53
Fibre (%)	10.93 ± 1.39	8.14	13.55
Ash (%)	2.04 ± 0.43	1.36	2.94
Other including carbohydrates (%)	12.31 ± 5.63	6.23	29.12
Energy (Kcal/100 g)	20.31 ± 3.73	11.68	28.87
Ripening index	83.70 ± 27.02	37.24	141.11
